# Practical Observations on Certain Diseases of the Chest, and on the Principles of Auscultation

**Published:** 1848-10

**Authors:** 


ART. IV.—*Practical Observations on certain Diseases of the Chest, and on the Principles of Auscultation.* By PEYTON BLAKESTON, M. D., F. R. S., etc. Philadelphia. Lea & Blanchard. 8vo. pp. 385.

It is now generally conceded, that diseases of the chest can only be correctly determined and discriminated by those who are practically acquainted with the principles of auscultation. So important is this art now considered in this country, that there is not a single medical school to be found, it is believed, in which it is not taught; and a course of medical instruction, into which this did not largely enter, would be deemed very imperfect and unsatisfactory. That the art is perfect, no one believes; that the conclusions derived from it, are sometimes erroneous, we all acknowledge; but that they are, for the most part, founded on truth, and furnish important rules for diagnosis and treatment, is universally admitted. True, we have some forms of disease for the detection or treatment of which no rules have as yet been laid down; this only proves, that additional facts are required for the establishment of other general principles, which may serve to prove the truth or fallacy of those already proposed. Hence it becomes the duty of those, who are favorably situated, to record and publish their observations; and hence, also, the value of reporting accurately, individual cases of disease, with their physical signs at different stages; and when fatal, the autopsic appearances. It is this feature in the works of Andral, Louis, and Abercrombie, which give them so much value in the eyes of medical men; and it is this same feature which stamps the present work with equal value.

The first fifty pages are devoted to a consideration of the "*Properties of Sound,*" in which the principles of auscultation are developed in a very elementary manner. To those whose preliminary education has been incomplete, and whose acquaintance with the laws of physical science is necessarily imperfect, this part of the work will prove extremely valuable; to others, it will not be necessary.

We pass over the chapters devoted to the auscultation of the *Sounds of Respiration*, of the *Voice* and the *Heart*; also, on the *Practice of Auscultation*, in which we discover nothing particularly new. The chapter upon the *Formation, Termination, and Causes of Thorcaic Aneurism*, abounds with useful hints and valuable cases, which do not however admit of condensative or complete analysis. On this subject the work of Hope is far the most satisfactory, and far in advance of any thing yet presented to the practitioner. An analysis of the thirty-seven cases presented by Dr. Blakiston leads to the following results:

1. No diagnostic sign was furnished by the character of the pulse, or by the presence of pulsations above or below the clavicles.

2. When a pulsation was seen and felt over a prominent point in the chest, it indicated the presence of a sacculated or mixed aneurism.

3. Purring thrill was only valuable as a sign of aneurism in conjunction with other signs.

4. A Systolic murmur, heard at a distance from the heart, even though it were not heard at the precordial region, only afforded evidence of the existence of aneurism when it was combined with other signs denoting the existence of a circumscribed tumor.

5. A double or diastolic murmur confined to one spot, at a distance from the precordial region, denoted the existence of a sacculated aneurism.

6. When a hollow murmur was heard, a dilated aneurism was present.

7. The intensity of aneurismal murmur was in a great measure proportioned to the force of the heart's action.

8. Aneurism of both kinds existed without the slightest trace of pulsation or murmur.

9. Aneurism arising within the sac of the pericardium were not indicated during lifetime by any characteristic signs.

In relation to the treatment of thoracic aneurism, we observe nothing but what may be found in all systematic treatises of this subject The indications, of course, are, the prevention of the increase, or rupture of a dilated aneurism; and 2nd. the obliteration of a sacculated aneurism by the deposition of fibrinous clot within it. The first indication is to be met by strengthening the walls of the pouch, or by diminishing the force of the current of blood, or by both means. The walls of the pouch may be strengthened by inducing inflammatory action in them, whereby lymph may be poured out between the coats. This might possibly be accomplished by a highly stimulating diet, but the dangers attending such a plan, are too great to be hazarded. An extremely poor diet is equally objectionable, as are also large and frequent bleedings, and the immoderate use of purgatives.

The author regards digitalis as a dangerous sedative agent in these cases, and prefers the poppy and hyoscyamus as equally efficacious, and far safer. The extract of belladonna, rubbed over the precordial region, is recommended for tranquilizing the actions of the heart, as superior to any other drug. Though mental and bodily repose are important, they are not to be carried to such extent as to derange the organs of digestion.

Where iron is indicated, the author recommends the *Tincture of the Sesquichloride,* with hyoscyamus. The treatment, in short, is to consist in the use of sedative and purgative drugs, a moderate amount of food of a nutritious, but not over stimulating quality, rest of mind and body, and the application of cold to the surface of the chest nearest the sac. The treatment, however, must be modified by the circumstances of the case, and according as the disease is of the dilated or sacculated variety.

There are 64 cases, fully detailed, illustrating the progress and terminations of chronic heart diseases; from which, the following flattering conclusions are drawn:—

1. A considerable amount of obstruction may exist at the aortic orifice of the heart, without seriously affecting the general health.

2. Mitral regurgitation is one of the most direct and frequent causes of pulmonary venous congestion.

3. Tricuspid regurgitation, is the most, direct, and almost constant cause of that engorgement of the vessels of the general circulation and its consequences, which originate with the heart.

4. Except in conjunction with regurgitation through the auriculo—ventricular orifice, hypertrophy of the ventricles in many cases rather assists the circulation, than promotes congestion.

With respect to the diagnosis of chronic heart diseases, we believe that cases often occur, in which it is next to impossible to determine the exact seat or amount of valvular derangement, notwithstanding all the aids furnished by modern research, and the refinements of some recent writers. Irregular action of the heart may arise from so many causes, that the practitioner is often baffled in arriving at a knowledge of the true one.— Thus, cardiac derangements may originate from dyspepsia, hysteria, and nervous irritability, hyperaemia, and anemia, without any organic change whatever. Organic causes, however, are always to be suspected and searched for, even when any of the above conditions exist. Dyspepsia, nervous irritability and hyperaemia are easily recognised. In anemia, a murmur is sometimes heard of the same character as that which occurs in aortic obstruction. It is seldom, however, persistent during a state of perfect repose; as, during sleep, upon gentle pressure, also, a similar sound may be detected on the course of the arteries of the neck. Under these circumstances, where the aortic murmur does not exceed that of a soft bellows sound, we may conclude that the cause of derangement is inorganic. Sometimes when the debility and irritability are very great, the heart becomes dilated to such an extent as to lead to tricuspid and mitral regurgitation.

But supposing we have arrived at the conclusion that the disease is of an organic nature, the next point to ascertain is, whether the difficulty exists in the contractile power of the heart, or the valvular apparatus, or both. If there is an increase of contractile power, it must be referred to hypertrophy of the left ventricle, indicated by a strong heaving impulse communicated to the hand, when laid over the cardiac region; the extent of precordial dullness will also be increased, more particularly, if, at the same time, the ventricles are dilated. The systolic sound, too, will be more muffled, and heard at a greater distance than usual from the precordial region. If there be a disease of contractile power, it must arise from attenuation and softening, perhaps from adhesion of the pericardium to the heart. If there be simple attenuation from dilatation, the extent of dullness over the precordial region is increased, and the sounds of the heart, although feeble, are sharp and clear, and are heard at a much greater distance than usual. If the ventricles are softened, especially if they are at the same time hypertrophied, both sounds are extremely feeble, and very much muffled and confused. We doubt whether there is any sign by which adhesion of the pericardium to the heart can be recognised.

With respect to *derangement of the valvular apparatus of the heart*, we have to ascertain, if possible, its *seat* and *nature*. If there be neither urgent dyspnoea, nor any signs of obstructed general circulation, we suspect the aortic orifice; but if very urgent dyspnœa be present alone, then we look to the mitral orifice, in expectation of finding regurgitation through it. If there be signs of obstruction to the general circulation, we shall expect to find tricuspid regurgitation, with, or without disease on the left side of the heart, according as the signs of pulmonary obstruction are present or not.

*Aortic Orifice.*—It is asserted by some writers that the seat of valvular murmurs is clearly indicated by their being heard with the greatest intensity at the exact spot on the surface of the chest which lies immediately over the orifices in which they are respectively produced. Thus disease of the aortic orifice would give rise to a murmur, the greatest intensity of which would be under the left edge of the sternum below the third rib. But very often we find the maximum of such sound considerably to the right of this point, especially if hypertrophy be present. It is far more important to notice the direction in which murmurs are propagated. Thus when the murmur is aortic, it can generally be clearly traced for a considerable distance up the course of the aorta, and in some cases, it is heard louder towards the arch of the vessel than over the seat of the aortic valves. When the disease is obstructive only, the murmur is systolic, and its strength and the quality of sound depend in a great measure on the contractile power of the left ventricle, and on the nature of the material with which the orifice of the vessel is obstructed. When aortic regurgitation takes place, the sound is either diastolic or double. The diastolic sound in such cases is often heard farther to the right side than the systolic sound.

*Mitral Orifice.*—The physical signs of mitral obstruction are not in general so well marked as those of aortic obstructions; in a great measure, because the size of the auriculo-ventricular orifice is so large, that a considerable amount of disease may exist without sufficiently narrowing it to give rise to sound. When a murmur is thus produced, it is of course diastolic; and as a general rule, it may be heard louder towards the apex of the heart than elsewhere, and also with great distinctness near the lower angle of the left scapula. It is often, however, extremely difficult by its seat alone to distinguish this murmur from that of aortic regurgitation; but as in the latter case, there would be visible arterial pulsations, and none in the former, the differential diagnosis is rendered tolerably sure. Some also insist on the difference in the pulse in these two cases; it being small, irregular and tremulous in mitral disease, and jerking and sharp in aortic regurgitation; our author, however, questions the value of this sign, and states that a great amount of obstruction may exist at the mitral valve in some rare cases, where, in consequence of the aperture being contracted to a very narrow slit, the stream passes noiselessly through it. It is not always possible, therefore, to detect mitral regurgitation by a murmur when it exists, and not unfrequently no murmur at all is engendered.

*Tricuspid Orifice.*—Obstruction very rarely occurs at this orifice. It is generally supposed that when regurgitation takes place in this situation a systolic murmur is produced; but that is by no means a certain sign. Very often we observe in such cases venous pulsations or undulations in the neck, more marked on the right than on the left side; Dr. Hope, however, thinks that they often arise from hypertrophy alone. The absence of venous pulsation by no means proves the absence of regurgitation. Their strength is rather a measure of the contractile force of the heart. Thus, if both ventricles are hypertrophied, a strong venous current will be met by an equally strong regurgitating current, the shock will be great, and the pulsations up the veins of the neck will be strongly marked. If both ventricles are softened or attenuated, a feeble venous current will be met by an equally feeble regurgitating current, and the thrill and pulsation will be slight, whilst the flow of venous blood will be as much impeded in the one case as in the other.

To sum up, we have then the following signs of valvular derangements:

Aortic Orifice—*Obstruction*—Systolic murmur traced up the course of the aorta, sometimes very prolonged. *Regurgitation*—Diastolic murmur running up the aorta, visible arterial pulsations.

Mitral Orifice—*Obstruction.*—Sometimes diastolic murmur at the apex of the heart, and at the lower angle of the left scapula, not up the aorta—without visible arterial pulsations—Pulmonary obstructions.

*Regurgitation.*—Sometimes, but not often, systolic murmur, heard at the apex of the heart, and at the lower angle of the left scapula, occasionally undulations between the second and third left ribs. Pulmonary obstruction—

*Tricuspid Orifice—Regurgitation.*—Seldom any murmur—venous pulsations of the neck, obstruction of the general circulation. Dropsy—

It is to be remembered that correctness of diagnosis is only valuable as it lays a foundation for treatment. With this in view, the chief endeavor of the practical physician will be to determine if organic disease of the heart is present, whether its contractile power is increased or diminished, and whether either or both of the circulations which result from it are obstructed. This will be accomplished with ease in most cases, although it may not be always possible to predict the exact state of each of the different valves.

The *Treatment* of chronic heart disease is very unsatisfactory. If there be no obstruction of the circulation, but evidences of hypertrophy of the ventricles, Dr. B. recommends occasional small bleedings, and opium, hyoscyamus and conium to quiet the action of the heart; and especially local frictions with the belladonna or opium ointment over the precordial region. It is useless to attempt diminishing the size of the heart, but patients treated on the above plan, often live many years in a state of comparative ease and comfort. Cold to the head habitually is found to prevent that cerebral congestion, so often dependent on such a state of the heart. To prevent valvular disease the author recommends mild mercurial frictions also over the precordial region, with a diet moderately stimulating, but sufficiently nutritious to sustain the strength of the patient. If the action of the heart be enfeebled, a tonic and sedative treatment is clearly indicated; such as the sesquichloride of iron, generous diet, combined with mental repose, and moderate bodily exercise.

If there exist both hypertrophy and disease of the valves, on the left side of the heart, it is important to ascertain, if possible, whether the structural changes of the valves, are of such a nature as to give rise to mitral regurgitation or not; for if such be not the case, then we must be very careful how we venture to control the action of the heart, because this increase of force has the effect of keeping up the circulation by counteracting the effects of mitral and aortic obstruction, and of aortic regurgitation. If, however, mitral regurgitation takes place, we must, if possible, diminish the energy of the heart's action, in the manner just pointed out; because dilatation of the right side is now more than ever to be dreaded, from the engorgement of the right ventricle produced by pulmonary congestion. When aortic regurgitation takes place, if the action of the heart be rendered slower by digitalis, the diastole is prolonged, and the effect of regurgitation is thereby increased. Nature often gives relief in these cases by promoting a copious secretion from the muçous membranes of the bronchial tubes. Hence benefit is derived from expectorant remedies, such as squills, with camphor, ether, &c. We should also aim to relieve pulmonary congestion by the occasional application of leeches or cups between the shoulders, and blisters to the chest. If, in addition to the signs of valvular disease, the action of the heart is feeble, of course the tonic treatment must be combined with the means above mentioned.

Cases in which there are symptoms of general obstruction, may exist either with, or without those of pulmonary obstruction; that is, without obstructions arising from an imperfect state of the valves on the left side of the heart. When both the pulmonary and general circulations are thus obstructed, the case is very formidable, and generally drawing to a close; yet when there are no signs of disease on the left side of the heart, although it may often be impossible to pronounce whether there is structural derangement of the tricuspid valves, or whether the orifice is simply dilated; yet we can discover important guides to treatment, according as chronic disease of the lungs is present, or as the force of the heart's action is increased or diminished.

The treatment is to be conducted according to the rules above laid down; but in addition, we have now to relieve, if possible, the engorgement of the capillaries, and remove the serum that has been poured out. This is to be done by increasing the secretions from the kidneys, bowels, or skin. The former plan is generally the most successful. In some cases, simple diuretics will succeed, such as squills, with nitric ether, acetate, or nitrate of potash, spirits of juniper, &c., with drinks made of cream of tartar, &c. Sometimes digitalis will succeed, when these fail. But to prevent its depressing effect on the system, which, in most cases is injurious, it must be carefully guarded with ammonia, wine, &c., when it proves a very powerful and safe diuretic. A good formula in such cases, is the following—infusion of digitalis, oss—spts. nit. ether, ℥ss, tinct. of squills, ʒii, comp. tinct. cardamom. ℥ss, acetate of potass., ʒiii, sesquicarb of ammonia, ʒss—of this an ounce should be taken two or three times a day.

The chapters devoted to circumscribed and chronic *pleurisy*, embrace many interesting cases, which do not admit of analysis; they may be consulted by the practitioner with great advantage. In the treatment of acute pleurisy, the author proscribes general bleeding, and relies chiefly in leeches, and frictions of the affected side with mercurial and opium ointment, after the following formula.—℞ ung. hydrarg, fort. ℥i—camphoræ, ʒss, pulv. opii. ʒi, misce. Between each friction, the chest is to be covered with large linseed-meal poultices, diluent drinks given, and aperient or diuretic medicines, singly, or in combination, according to circumstances. On the subsidence of the acute symptoms, the friction is to be discontinued, and the removal of the effused fluid promoted, if necessary, by blisters and diuretics. Under this treatment, D. B. states that no case of primary, uncomplicated pleurisy, terminated fatally, however acute the attack: but when other organs were affected, or when the pleurisy appeared in the course of some chronic disease, several cases terminated in death.

In the treatment of chronic pleurisy, Dr. B. relies chiefly on mild iodine, and mercurial frictions, with opium, if pain is present, and cream of tartar as a drink. If these means fail, blisters are applied, and the surface dressed with mild mercurial ointment, and stronger diuretic medicines given, light nutritious food, and mercurial tonics are also enjoined, the latter at a later period of the disease. In the more advanced eases, large blisters are early applied, followed by mercurial frictions, supporting the strength of the patient at the same time, by wine, quinine, ammonia, and beef tea. These means were found to diminish, rather than increase the fever. In one case only out of 90, was paracentesis resorted to. Louis states that he has never seen a case of pleurisy, in which paracentesis was indicated.

*Plastic Pneumonia.*—Under this name, Dr. B. treats of ordinary pneumonia, or inflammation of the parenchyma of the lungs, the results of which are known under the names of *red and gray softening or hepatization and induration.*

There are two forms in ordinary acute pneumonia, one produced by congestion and inflammatory swelling of the vessels, and the other by the deposition of lymph. Dr. B. supposes that there is a form of pneumonia, which he calls *plastic,* in which lymph is deposited, with little or no fluid, and this may occur either in an aggregated or diseminated form, and the result chiefly of low, chronic inflammation. Cases are given in illustration, to which we can only refer.

*Treatment of Pneumonia.*—In this country, there is but little difference of opinion as to the proper treatment of pneumonia. In general, bleeding is well borne, and attended with the most decidedly beneficial effects. Some practitioners, however, attempt to manage the disease by diaphoretics, blisters, &c., without resorting to depletion by the lancet or leeches; cases treated in this way, are more protracted, and also more frequently terminate fatally. Regard, undoubtedly, is to be paid to the opposite forms which it assumes, and the difference in the anatomical character of its several stages. But we speak of the disease, as it usually prevails, and its early stage. A severe attack of pneumonia, if not treated early by active depletion, will soon assume a typhoid type, in which bleeding is out of the question. Hence we reason, that we have so much of *typhoid pneumonia* in the practice of some physicians; the typhoid symptoms being the result of improper treatment.

Dr. B. extols the plan of Laennec, in treating pneumonia, that is, by giving large doses of antimony, such as six grains in the course of 24 hours, gradually increased to one scruple. Our author states that he has employed this treatment for the last 12 years, and believes it suitable, with certain modifications, in individual cases, to every form and stage of primary pneumonia. Of 61 cases thus treated, in which the disease was simple, three died, and fifty eight recovered. He thinks mercury may be useful in the typhoid forms of the complaint, but that its direct influence in arresting acute inflammations of parenchymatous organs, is more than doubtful, although he admits its value in similar affections of serous and sero-fibrous tissues. We have made full trial of the different modes of treating pneumonia, in an extensive dispensary practice, and we confess we are not convinced that the tart. antimony practice, will prove as successful, as the depletory by general and local bleeding. In our hands it has not proved satisfactory, and we believe this is the experience of most practitioners. In typhoid cases, mercury, opium, and counter irritation, with wine, quinine and ammonia, are recommended by Dr. B.

*Phthisis Pulmonalis.*—This term is very properly limited by Dr. B. to cases, where tubercular matter is found in the lungs. Under the microscope, tubercle is seen to be composed of three elements:—

1. An amorphous, transparent stroma, which reacts chemically like coagulated fibrin.—

2. Minute granules, some of which consist of at, some of protein compounds, and others of earthy salts.—

3. Corpuscles, generally about one third the size of pus-cells—and which are imperfectly developed cells. In recent firm tubercle, the amorphous stroma and cytoblasts predominate; the latter being often closely clustered together. In soft tubercles, they are generally further apart, and less numerous, whilst the granules are greatly increased. Tubercular matter, therefore, contains the elements of nutrit ion in a degraded form, and may be considered as a product of abnormal nutrition, in which an abortive attempt at organization has taken place. As the same kind of matter is secreted in some forms of typhoid fever, and in scrofula, phthisis is now considered, like these diseases, to depend on a peculiar derangement of the constitution, called the tubercular diathesis. We know not in what this consists. Some pathologists state that in the early stages of the disease the blood contains less fat than in health; Andral has detected an increase of fibrin relative to the number of blood corpuscles; but this change belongs to all inflammatory diseases. Simon supposes that this change may be accounted for by the acceleration of the circulation which usually takes place soon after the disease has set in; by which means the blood corpuscles are consumed more quickly in the lungs than they are formed in the chyle, thus causing a disproportion between them and the fibrin. The tubercular diathesis is evidently connected with an asthenic state of the system.

*Development.*—Tubercular matter is never seen in any other than the solid form. Vogel supposes that it is secreted from the capillary vessels in the fluid form, and that it afterwards fills up the interstices of the tissues in a manner too perfect to be accomplished by any substance that was not originally fluid. It may be considered to be formed from an amorphous fluid blastema, like pus and other morbid products, and in three ways.

1. Directly from the fluid blastema, as a single product. Then the smallest grains of tubercular matter have, not unfrequently, the same appearance and ultimate structure as the larger masses, being yellow and opaque, and having the granular elements numerous in proportion to the corpuscles.

2. Other products may be simultaneously formed with it from the same blastema. Such as Lymph, &c.

3. It may be preceded by the formation of other products of abnormal nutrition, such as a gray semi-transparent matter, which occurs under three forms:

1. Small roundish tumors, called granulations. 2. Masses, surrounding cavities. 3. Considerable sized masses inclosing a few yellow tubercles. The first two are very common. Louis states that he hardly ever met with a case of yellow tubercles without the presence of gray granulations. The gray masses are doubtless the result of chronic inflammation. They consist of coagulable lymph, incapable of reaching the degree of organization attained by that thrown out in more active inflammation, and occurring in a sthenic state of the constitution. The gray granulations are considered by Louis and Laennec as tubercular; the first stage of yellow tubercle. Carswell regards them as formed of inspissated mucus in the air-cells. frequently inclosing a portion of tubercular matter. Andral considers them as air-cells thickened by inflammation, as do also, Alison, Williams, and others. Vogel, Lebert, and Bennett describe them as composed of the same elements as yellow tubercle. Dr. B. considers the gray matter to be an imperfectly organized substance, which, in the absence of the tubercular diathesis, may remain stationary, or may rise slightly in the scale of organization, but which, under opposite circumstances, may descend in the scale, and be converted into, or displaced by tubercular matter. In this sense it may in some cases be the first stage of tubercle, but not a stage, as supposed by Louis and Laennec, through which tubercles must necessarily pass before acquiring their own peculiar character, nor must it necessarily be followed by tubercle. How yellow tubercle is developed in the midst of the gray matter, is unknown. Whether it is deposited as a nidus, in the same manner as earthy salts are deposited in atheroma, or whether the gray matter is transformed into it as lymph is converted into pus. Both suppositions may be reconciled with our present knowledge. Thus, if we suppose tubercle to be deposited in the gray matter, this process must be the result of an increased tubercular diathesis, whereby a blastema highly charged with tubercular matter is thrown out. But if the gray matter after remaining a certain time is transformed into tubercle, it must be in consequence of diminution in the activity of the metamorphosis of tissue, by the force of which it had been hitherto kept from descending in the scale of organization. It is well known that normal tissue cannot be directly transformed into pathological formations, which arise out of an amorphous blastema. Gray matter is not, however, normal tissue, but an imperfectly organized substance. Both tubercle and gray matter are protein-compounds, of the transformations of which we are as yet ignorant, so that there is nothing improbable in the notion that the one is transformed into the other. It is probable that the deposition of tubercular matter in the lungs is always preceded by local congestion. Vogel considers it to result from the same causes as fibrinous dropsy generally, and to be preceded by local hyperæmia of the precipitating capillaies. Andral states that the pre-existence of congestion may be admitted, but cannot be demonstrated. As to the vexed question, whether the action which accompanies the deposition of tubercle be inflammatory or not, pathologists are divided in opinion. If by the term inflammation, be understood pain, heat, redness and swelling, or the presence of lymph or pus, then tubercle is not inflammatory. If, on the other hand, we consider the essential phenomenon of inflammation to be an increased exudation of blood-plasma, then tubercle must be regarded as an inflammatory product. At any rate its deposition depends on a peculiar asthenic condition of the system, called the *tubercular diathesis*, and is preceded by *local hyperemia.*

*Causes of Tubercular Phthisis.*—These may be regarded as two-fold—the one including the tubercular diathesis; the other favoring the development of tubercles in the lungs, after this constitutional derangement has taken place.

Of the former class of causes, Louis has shown that the greater number of these have been admitted on insufficient data: and in this conclusion, our author also argues. M. Louis admits as causes of the tubercular diathesis, *lymphatic temperament, female sex, and continued febrile action*: he rejects as special causes, bad food, impure air, depressing passions, and other debilitating circumstances; but allows them to be causes of a variety of diseases, of which, phthisis is one. He excludes *climate and temperature, trade and occupation, delicacy of constitution, and inflammations of the thoracic viscera.* Dr. B. states that his observations fully confirm the influence of *temperament, sex, and continued febrile action*: but they further tend to prove, that some causes, which are only allowed by Louis to act generally, are, in fact, special causes of phthisis. These are *mental and physical depression*. Among these latter, the *Influenza* of 1837, occupies an important rank. This disease was not accompanied by long continued febrile action, but it was characterized by extreme depression of the nervous system. Dr. B. states that a great number of cases of phthisis seemed to owe their origin to an attack of this disease; and occurred in persons who had previously been in good health, and presented no traces of strumous diathesis. *Chlorosis*, also, is often followed by phthisis; and the same remark will apply to gonorrhea, and syphilis, which the author attributes to mental depression, consequent on the disease. The influence of hereditary transmission is stated not to have been strongly marked, in the cases which fell under the writer's observation. In regard to the influence of trade and occupation, Dr. B. states that facts go to prove, that the development of the tubercular diathesis is not induced by any particular mode of employment, but that, amongst those in whom it already exists, the deposit of tubercular matter in the lungs is sometime precipitated. The results of pleuritis and pneumonia, as observed by Dr. B., tend fully to confirm the opinion of Louis, that inflammation of the throracic viscera do not induce the tubercular diathesis. It is obvious, however, that inflammation of any organ may induce constitutional derangement, by impairing its functions, and thereby interfering with the formation or purification of the blood, to which the proper performance of such functions may be essential. In this way, inflammation of an organ concerned in the process of primary assimilation, may indirectly favor the development of the tubercular diathesis.

*Exciting Causes.*—All causes which determine the blood to any particular parts of the body, must, says Dr. B., facilitate the deposition of tubercle in those parts, if the tubercular diathesis previously exist. The principal are *age, cold applied to the surface of the body, suppression of the menses, certain forms of fever, inflammation of the thoraric viscera.*

*Termination.*—Some writers have contended that phthisis pulmonalis, always necessarily terminates fatally; others, admit the possibility of a recovery, but believe such cases to be rare; while others still, maintain that cases of recovery from the disease are very frequent. It is well observed that if solid whitish nodules, of the consistence of condensed cellular tissue or fibro-cartilage in the lungs, are to be considered as denoting the previous existence of tubercular deposit, the disappearance of tubercle must be allowed to be a very common occurrence. But it is worthy of notice, that these appearances are found in the lungs of persons who have never exhibited the slightest symptoms of phthisis, and it is highly probable that they are often the result of plastic pneumonia. It is the opinion of Andral, that unless a bronchial ramification can be traced into these substances, the pre-existence of a cavity can not be deduced. But this is rarely the case. Calcareous matter is not unfrequently found in the lungs; but it has not yet been demonstrated, that these deposits have always been preceded by tubercular matter. As we find calcareous deposit taking place around fibrinous concretions within the heart, and around coagulated lymph in other parts of the body, so the same may take place in the lungs, without such lymph being connected with tubercles. The fact, however, is well established, that tubercular matter may be deposited in the lungs, and be replaced or surrounded by matter of cellulo-fibrous, fibro-cartilaginous, or calcareous consistence, without having inflicted any notable injury on the health; but whether recovery ever takes place after decided signs of tubercular deposit have been manifested in life, remains to be proved. Many cases have occurred, where, after unequivocal signs of solid deposit have been manifested at the summit of one lung, either the physical signs of disease have disappeared, and complete recovery taken place, or else, in which some chronic catarrh alone has remained, &c. But there is no evidence in these cases, that tubercles have been deposited. The physical signs may have been owing to consolidated lymph. That tubercular disease of the lungs is often arrested, or prevented by appropriate treatment, no one can doubt. Cases of this kind are given by our author.

*Treatment of Phthisis.*—The treatment of this disease may be classed under two divisions; 1st *empirical*, consisting of certain *specific* remedies; 2d *rational*, founded on what we know of its pathology, causes, &c, We need not dwell upon the specifics recommended in former years, as they have not stood the test of experience, and are obsolete. *Naptha* has more recently been introduced as a specific, but is not likely to meet with a more fortunate result than the former. It was employed in 50 cases by Dr. B where there were evidences of cavities in one or both lungs, but without the slightest benefit. In 50 cases where there were evidences of solid deposit in the summit of one lung, or both, it seemed to improve the tone of the digestive organs in some cases, but, on the whole, the disease was not arrested in a single instance, and tubercular softening came on as rapidly as in cases where it was not given.

The author has been more successful with *cod liver oil*, having, also, used it in 100 cases, 12 of which, in the incipient stage decidedly improved; confirmed cases were greatly relieved; in 5 it was obstinately rejected from the stomach; in 11 it purged, and could not be taken. On the whole, although not a specific, yet it may be usefully given in certain cases, conjoined with a judicious system of regimen and diet.

Three plans of treatment have been employed in phthisis, viz: the *antiphlogistic*, the *expectant* and the *tonic*. The antiphlogistic was advocated by Broussais, and subsequently by Dr. Stokes, who endeavors to prove that it is the only mode of arresting the disease in its early periods. In carrying out this plan, bleeding, general and local, is freely employed at the outset of the disease, followed by counter-irritation and mercury. This treatment is founded on the erroneous belief, that tubercular deposit in the lungs is the result of acute inflammatory attacks of that organ; a doctrine now generally set aside for that advocated by Louis, and others.

The *expectant* treatment is now resorted to by a large class of medical practitioners; consisting in a regulated diet, attention to exercise, and bathing, with the occasional use of expectorant medicines, etc. Its chief characteristic lies in the means which are taken for preventing exposure to cold, and guarding against irritations of the lung.

The *tonic* treatment has also numerous advocates among the ablest members of the profession; consisting in regularity of habit, appropriate exercise, pure air, suitable food, the use of bitters and chalybeates, cold bathing, etc. The chief object of this treatment is directed to improve the constitution generally, and thus remove the tubercular cachexia, at the same time not overlooking the local disease, which is, for the most part, treated with sedative medicines, and very moderate local depletion, occasionally by counter-irritation, and mild mercurials. But these remedies are to be so selected and administered, as not materially to lower the system, and thus interfere with the primary object of the treatment.

It is now generally conceded that the deposition of tubercle in the lung, is invariably preceded by an altered state of the constitution generally; and that, consequently, the principal object of treatment should be the improvement of the constitution. This constitutional derangement, moreover, is of an asthenic character, a kind of chronic, typhoid state. It is true, that in a certain stage of the disease, the condition of the blood resembles that found in inflammatory affections, but then such inflammations are as frequently of an asthenic as of a sthenic character, and do not call for antiphlogistic measures. The nature of the disease, then, fully proves, that the invigoration of the system by nutritious and tonic regimen and diet, is the best means of fulfilling the primary object of treatment, the correction of the tubercular diathesis.

The known causes of the disease indicate the same course of treatment. The general causes are all of a debilitating character; and the same may be said of the special causes, moral and physical depression, febrile attacks, uterine derangement, &c., &c., all of which require a tonic and alterative course. It is true, there is great irritability often associated with this debility, demanding the use of sedative remedies in connection with tonics. By this means the irritability of the lungs is relieved, while, at the same time, the general strength is not diminished, In applying these principles to the prevention, removal and palliation of phthisis, circumstances occasionally occur, which require that the plan of treatment should be somewhat modified; such as the use of counter-irritants, and topical bleedings.

*Prevention.*—The prophylactic treatment of consumption can not be commenced at too early an age, whether there be a predisposition to the disease or not. Our author recommends that where the complaint has existed in the mother's family, or where she is in a delicate state of health, a wet-nurse should be provided, and maintained in good health by nutricious diet, regular exercise in the open air, frequent ablutions of the baby, and living in large, well-aired apartments. Meat-tea is to be allowed the infant, even at the breast. Should it not acquire the average degree of plumpness, or its flesh be pale and flabby, under its judicious use, infants will often be restored from a state of marasmus to vigorous health. Too much can not be said in favor of frequent exposure to the open air; the clothing should be moderately warm, thin flannel being next the skin, so arranged as to leave the body unconstrained, and to allow of the free movements of all its parts. The surface of the body is to be habitually washed and rubbed; cold water is generally to be employed, though at certain seasons the chill should be taken off. That which is applied to the head should be always cold, in order to prevent congestion.

The restlessness, and other symptoms of cerebral disturbance which occur about the period of dentition, are best relieved, according to our author, by mild medicines, such as a few drops of syrup of poppies or morphia, at the same time favoring the passage of the teeth by incision of the gums; and he remarks, that Louis has shown the groundlessness of the dread entertained by many of the sedative medicines producing congestion of the brain, or arresting the development of the intellectual faculties of infants. While these means are pursued, we are to watch closely any indications of a tendency to tubercular deposit in any part of the body.

At this period of life, the brain and its membranes, the peritoneum, and the glands of the mesentery and neck, are more often the seat of tubercular deposit. When the brain is the part affected, evidences of meningitis are present, and this is to be treated by mercurial purging, rather than general or local bleeding. Tubercular peritonitis will often be relieved by mercurial and opium frictions combined with mild nutritive diet, so prepared as to be easily digested. Tuberculization of the glands of the mesentery, causing marasmus, is to be treated by a combination of alkaline and alterative medicines, with some mild sedative; uniting farinaceous food with the liquid parts of animal flesh—iodide of potassium and cod liver oil, are the only remedies recommended in these cases. The same general plan is advised in combating the ordinary acute attacks of childhood and youth, employing antiphlogistic measures as sparingly as possible, and trusting rather to mercury and antimony as depressants. At the age of puberty, attention is to be directed to the generative system. Cautions given as to indelicate practices, like masturbation. Too close application to study, and sedentary occupations are to be carefully guarded against, while out-door exercise is to be constantly attended to. By these measures the integrity of the digestive organs will be preserved, and while these perform their healthy functions, there is slight danger of an invasion of phthisis.

In the advanced stage of the disease, certain symptoms occur, which it is important to be able to palliate at least, if we can do no more. To allay vomiting, iced drinks, opium, liquor potassæ, hydrocyanic acid, and naptha are recommended; diarrhœa, generally dependent on ulceration, is to be combatted by starch and anodyne injections, kino with quinine, &c. Cough will be relieved by morphia; and when convulsive, by small doses of hydrocyanic acid. When accompanied by excessive secretion from the bronchial tubes, the decoctiou of senega is often very serviceable. Tannin is recommended for night perspirations when other means fail. The dyspnœa is sometimes greatly relieved by nitric ether and sal volatile. Inhalation has not been found very useful in the hands of the author. In this respect our own experience leads us to a more favorable estimate of its merits.

We have thus given a pretty full analysis of Mr. Blakiston's work; which, although not strikingly original, is nevertheless characterized by good sense, and sound, practical judgment. The cases, which are very numerous, are succinctly detailed, and highly instructive. We cheerfully bear testimony to the practical value of the work, and trust it may meet with a wide circulation.

